# Selection for chloroquine-sensitive *Plasmodium falciparum* by wild *Anopheles arabiensis* in Southern Zambia

**DOI:** 10.1186/1475-2875-12-453

**Published:** 2013-12-19

**Authors:** Sungano Mharakurwa, Mavis Sialumano, Kun Liu, Alan Scott, Philip Thuma

**Affiliations:** 1 The Malaria Institute at Macha, PO Box 630166, Choma, Zambia; 2 Department of Molecular Microbiology & Immunology, Johns Hopkins Malaria Research Institute, Johns Hopkins Bloomberg School of Public Health, 615 N Wolfe Street, Baltimore, MD 21205, USA

## Abstract

**Background:**

The emergence of parasite drug resistance, especially *Plasmodium falciparum*, persists as a major obstacle for malaria control and elimination. To develop effective public health containment strategies, a clear understanding of factors that govern the emergence and spread of resistant parasites in the field is important. The current study documents selection for chloroquine-sensitive malaria parasites by wild *Anopheles arabiensis* in southern Zambia.

**Methods:**

In a 2,000-sq km region, mosquitoes were collected from human sleeping rooms using pyrethrum spray catches during the 2006 malaria transmission season. After morphological examination and molecular confirmation, vector mosquitoes were dissected to separate head and thorax from the abdominal section, followed by PCR screening for *P. falciparum* infection. Human residents of all ages were tested for *P. falciparum* parasitaemia by microscopy and PCR. *Plasmodium falciparum* infections were genotyped at the chloroquine resistance-conferring amino acid codon 76 of the *Pf*CRT gene, using PCR and restriction enzyme digestion.

**Results:**

In the human population there was nearly 90% prevalence of the chloroquine-resistant *Pf*CRT K76T mutant, with no significant differences in polymorphism among smear-positive and smear-negative (submicroscopic) infections (p = 0.323, n = 128). However, infections in both abdominal and salivary gland phases of the *An. arabiensis* vector exhibited wild type K76-bearing parasites with up to 9X higher odds (OR (95% CI): 9 (3.7-20.2), p < 0.0005, n = 125), despite having been acquired from humans within a few weeks.

**Conclusions:**

*Anopheles arabiensis* selects for wild-type K76-bearing *P. falciparum* during both abdominal and salivary gland phases of parasite development. The rapid vectorial selection, also recently seen with antifolate resistance, is evidence for parasite fitness cost in the mosquito, and may underpin regional heterogeneity in the emergence, spread and waning of drug resistance. Understanding the nature and direction of vector selection could be instrumental for rational curtailment of the spread of drug resistance in integrated malaria control and elimination programmes.

## Background

The emergence and spread of parasite drug resistance, especially *Plasmodium falciparum*, continues to pose a major obstacle for malaria control and elimination [1,2]. *Plasmodium falciparum* has repeatedly proven ability for mounting resistance to any anti-malarial drug regimen upon wider use, including current ones [2-7]. To avert potential resurgence owing to treatments that are no longer effective, not only are new anti-malarials needed, but also public health strategies that will minimize or delay drug resistance escalation [8,9].

There are only a few strategies that have been rationalized and adopted to date, namely, avoidance of sub-therapeutic doses [10,11], combination therapy, especially with artemisinin and its derivatives [12], and focused screening and treatment, which was recently started in Southeast Asia in an effort to stem the global spread of emerging *P. falciparum* tolerance to artemisinin [13]. So far *P. falciparum* evidently evades all these approaches [14,15].

To develop effective strategies for containment of drug resistance, an understanding of the epidemiological factors that govern the emergence and spread of resistant parasites in the field is paramount. Theoretical arguments [12,16], although sometimes conflicting [13,14], and a review [15], have posited an association between malaria transmission control and levels of *P. falciparum* drug resistance. Meanwhile, field studies have shown an unexpected but decisive association between mosquito control and the prevalence of *P. falciparum* drug resistance [17,18]. The prevalence of *P. falciparum* dihydrofolate reductase (DHFR) wild-type (antifolate drug-sensitive) alleles increased in Tanzania when insecticide-treated nets (ITNs) were implemented [16]. In an area of Zimbabwe, the introduction of indoor residual insecticide spraying (IRS) was associated with a four-fold reduction in the odds of chloroquine therapeutic failure after four years [17], despite concurrent drug pressure. Cessation of the IRS was linked to a four-fold rebound in odds of chloroquine resistance after another subsequent four years [17]. In contrast, Shah *et al.* found no significant relationship between sustained ITN use and the prevalence of chloroquine or antifolate drug resistance in western Kenya [19].

What remains unclear is why drug resistance associates with mosquito control in some areas but not others. Similarly, why does the removal of drug selection pressure result in decisive re-emergence of sensitive parasites in some areas [20-25] and yet other locations show slow recovery or fixation [26-29]? A recent study suggests that mosquito vectors may play a role in determining field levels of drug-resistant *P. falciparum*[30] and thus constitute an additional governing factor in drug resistance epidemiology*.* This means that mosquito control may influence parasite drug resistance indirectly, through impact on vector species that select on drug resistance polymorphism. Highly contrasting *P. falciparum* antifolate drug resistance polymorphisms were found in human and mosquito hosts that seemed to suggest strong selection against pyrimethamine resistance mutants by the *Anopheles arabiensis* vector [30]. The current study documents *An. arabiensis* selection on the chloroquine resistance-conferring polymorphism of the *P. falciparum* chloroquine resistance transporter (PfCRT) gene[31].

## Methods

### Study area and population

The study was based in a 2,000-sq km vicinity of the Malaria Institute at Macha (MIAM) and Macha Mission Hospital, a 208-bed, rural, district-level hospital in Southern Province of Zambia. The area has an altitude of 900–1,000 m above sea level and historically experienced hyperendemic malaria, which is transitioning to hypo-endemicity following scale-up of ITN and artemisinin combination therapy (ACT) interventions since 2004. The resident population is approximately 160,000 and comprises primarily subsistence farmers of the Batonga communities. As with the rest of Zambia, chloroquine, the former first-line treatment for malaria for many years, was suspended from use in 2003 and replaced with artemether-lumefantrine.

### Design

The study was a cross-sectional survey. *Plasmodium falciparum* chloroquine resistance polymorphism was determined in humans and local *An. arabiensis* vector mosquitoes.

### Parasite DNA sample collection

#### Mosquito phase

Following prior appointment with the resident communities, whole mosquitoes were collected from human sleeping rooms by pyrethrum spray catches in the morning from 06.00-10.00 [30].

### Human phase

Through prior arrangement, willing community residents of all ages assembled at existing central meeting points, during the afternoons of the same day as pyrethrum spray catches, for malaria screening by microscopy as previously described [30]. Parasite DNA samples were simultaneously collected as filter paper dry blood spots (DBS) from the same finger-prick used to prepare microscopy blood films.

### Parasite DNA extraction

#### Mosquitoes phase

The mosquitoes collected from sleeping rooms of the resident population were identified morphologically, with confirmation by sibling species complex differentiation PCR for *Anopheles gambiae s.l.* and *Anopheles funestus s.l.* as previously described [30,32]. Parasite DNA was extracted separately from the mosquito abdominal and salivary gland phase using a modified chelex protocol [33].

#### Human phase

Parasite DNA was extracted from DBS samples using the regular chelex method [34].

#### Assays for *Plasmodium falciparum* drug resistance alleles

*Plasmodium falciparum* infections were genotyped for the chloroquine resistance-conferring K76T mutation in the P*f*CRT gene using nested PCR and allele-specific restriction enzyme digestion [31,35].

### Ethical considerations

The study was approved by the Johns Hopkins IRB and the University Of Zambia Research Ethics Committee (UNZAREC) as part of an ongoing geographical and demographic reconnaissance study of malaria.

## Results

### Prevalence *Pf*CRT K76T in human malaria infections

**Figure 1:**
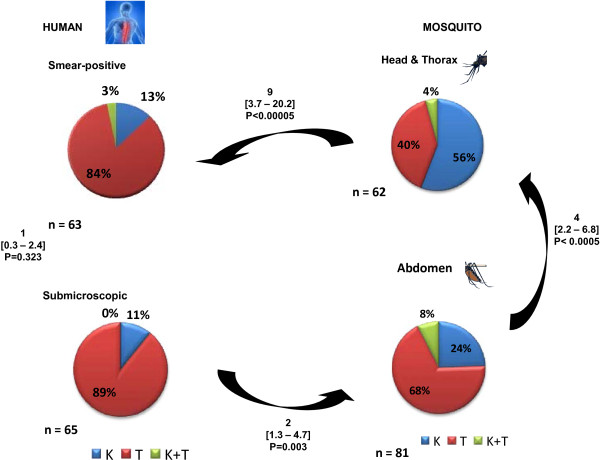
PfCRT K76T polymorphism in human and mosquito phases of infection (interspersing numbers are odds ratios (95% CI)).

From a total of 2,278 residents screened, 128 *P. falciparum* infections were genotyped for *Pf*CRT-76 polymorphism (Figure 1). In the humans, the prevalence of chloroquine-resistant K76T-bearing *P. falciparum* was virtually 90%. No significant differences in the K76T single nucleotide polymorphism (SNP) were observed among microscopy-positive and microscopy-negative (submicroscopic) human malaria infections (Figure 1).

### Prevalence *Pf*CRT K76T in mosquito malaria infections

Of 753 vector mosquitoes captured from human sleeping rooms, 745 (99%) were *An. arabiensis*, the remaining 8 (1%) being *An. funestus*. Despite having been acquired from humans within the previous ten days, *An. arabiensis* mosquito abdominal infections exhibited more than twice the proportion of wild-type (K76-bearing) *P. falciparum* that is sensitive to chloroquine (Figure 1). The proportion of K76-bearing *P. falciparum* was more than five times higher in the mosquito salivary gland phase than in humans, despite having been acquired from the latter within the previous few weeks defining the natural life span of a mosquito in the wild (Figure 1). Only one of the eight *An. funestus* was found positive for *P. falciparum* infection, which was in the abdomen and bore *Pf*CRT 76 T.

## Discussion

Data from the present study point to a strong natural selection for chloroquine-sensitive *P. falciparum* by *An. arabiensis* during both the abdominal and salivary gland phases of development. Within the short mosquito phase of the parasite, lasting only a few weeks, *P. falciparum* infections in salivary gland stage exhibited four-fold and nine-fold higher odds of chloroquine-sensitive parasites than abdominal and human phase infections, respectively. Thus, there appears to be two distinct and sequential stages of this “vectorial selection” for wild type *P. falciparum* in the mosquito host.

The observed data concur with earlier findings showing *An. arabiensis* selection against pyrimethamine resistance mutants [30] in the same area. Owing to low numbers for *An. funestus*, it could not be determined whether this vector exerts similar selection as seen in *An. arabiensis*. Nonetheless, in a Tanzanian study by Temu *et al.*[36], *An. arabiensis* appeared to consistently carry a lower proportion of PfCRT 76 T mutants than both *An. funestus* and *An. gambiae s.s*.

The mechanistic basis for the “vectorial selection” on drug resistance polymorphism is not clear from a cross-sectional field survey. However, fitness cost is a possible explanation. Drug resistance mutations, including *Pf*CRT, are known to bestow altered biological fitness on the parasite, which has so far been reported *in vitro,* in humans [37-42] and more recently in mosquitoes [30]. Mosquitoes have been shown to mount effective immune clearance of malarial infection, hence bottlenecking parasites during mid-gut as well as salivary gland invasion [43,44]. Presumably, less fit mutated parasites are largely eliminated during these successive survival-limiting stages of parasite development in the mosquito. Mosquitoes are also the definitive host where parasite genetic recombination takes place that can break down resistance haplotypes.

On the other hand, mid-gut microbiota have been reported to have direct anti-malarial properties [45-47] that could possibly impose selection on *P. falciparum* in the mosquito. Differences in gametocytogenesis [48,49] between mutated and wild type parasites may be another possible reason for selective parasite survival in the vector. However, both the mid-gut microbiota and gametocytogenesis would not explain the strong selection for K76 observed between abdominal and salivary gland phases of the parasite, which was similarly observed with antifolate resistance polymorphism [30]. Thus, a general fitness cost of resistance in the mosquito appears the most reasonable hypothesis.

Irrespective of the underlying mechanisms, the results observed in the present study appear to lend further evidence that vector mosquitoes may play an important role in drug resistance epidemiology [30]. It is hypothesized that the vectorial selection contributes to recovery of chloroquine-sensitive malaria parasites following withdrawal of drug use, as observed initially in Hainan Province of China [50] and subsequently Malawi [23,24] and other areas, including the site of the current study (Sialumano *et al.* in prep). Such natural vectorial selection is in concert with observations by Laufer *et al.* that re-emerging chloroquine-sensitive *P. falciparum* are pre-existing diverse parasites that survived the chloroquine era, rather than a new invading strain [51]. Vectorial selection on parasite drug resistance polymorphisms would also appear to explain the field observations of association between mosquito control and prevalence of *P. falciparum* drug resistance [17,18], and may underpin regional differences in susceptibility to the emergence and spread of drug resistance, depending on relative survival of mutated parasites in the local vector species. To better understand the possible role of the vector mosquitoes in anti-malarial drug resistance epidemiology, more field studies and controlled laboratory experiments are needed.

## Conclusions

*Anopheles arabiensis* selects for wild type (K76-bearing) *P. falciparum* during the abdominal and salivary gland phases of parasite development. The vectorial selection is evidence for *P. falciparum* drug resistance fitness cost in the mosquito and may underlie regional heterogeneity in the emergence, spread and waning of drug resistance. Understanding the nature and direction of vector selection could be instrumental for rational curtailment of the spread of drug resistance in integrated malaria control and elimination programmes.

## Competing interests

The authors declare that they have no competing interests.

## Authors’ contributions

SM was the principal investigator who initiated, designed and ran the study, followed by data analysis and writing of the manuscript. MS performed laboratory assays and reviewed the manuscript. KL reviewed and contributed to the manuscript. AS provided essential technical input and reviewed the manuscript. PT was the overall advisor and reviewed the manuscript. All authors read and approved the final manuscript.
